# Supplementary material for: Binding Mechanism of CD47 with SIRPα Variants and Its Antibody: Elucidated by Molecular Dynamics Simulations
Source: Molecules. 2023 Jun 7;28(12):4610. doi: 10.3390/molecules28124610 (PMC10304963; doi:10.3390/molecules28124610)
Supplement: Supplementary file 1 [file molecules-28-04610-s001.zip › molecules-2413013-supplementary.pdf]

Supporting Information

Binding Mechanism of CD47 with SIRPα Variants and Its Antibody: Elucidated by Molecular Dynamics Simulations

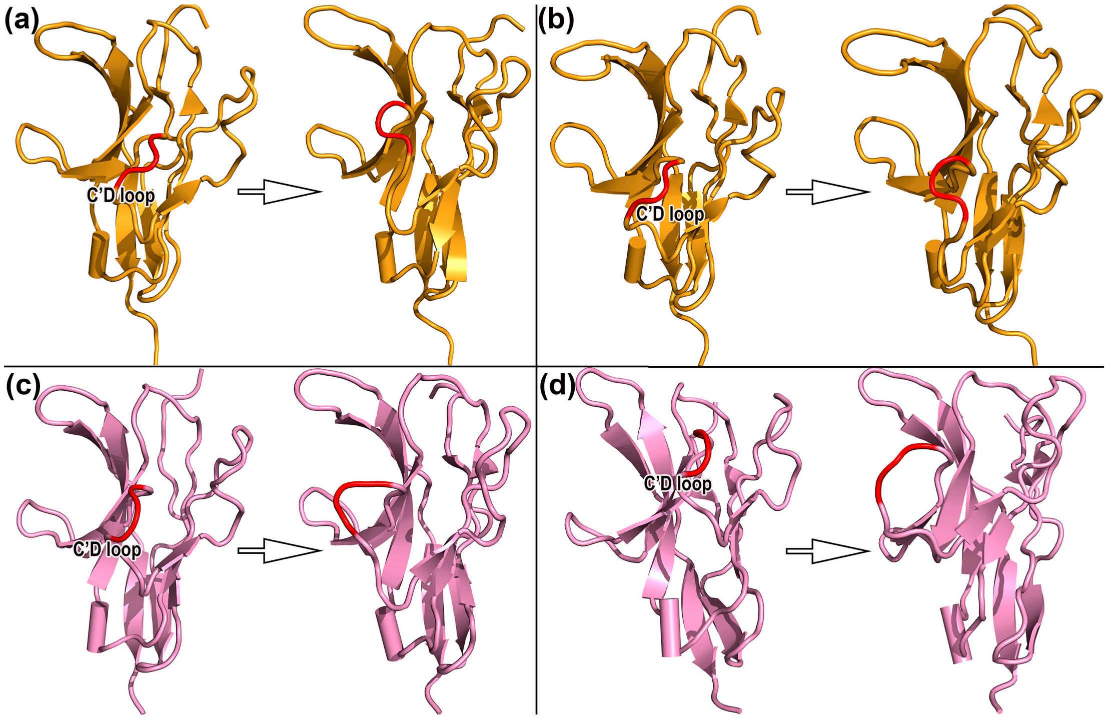

Figure S1. Dynamical changes of the C′D loops throughout the simulations. (**a**) SIRPαv1 in simulation2, (**b**) SIRPαv1 in simulation3, (**c**) SIRPαv2 in simulation2, (**d**) SIRPαv2 in simulation3. The left half of each figure shows the structural state at the beginning of the simulation, and the right half shows the equilibrium structural state at the end. The C′D loops are important components of the critical groove regions on the binding interfaces of SIRPαv1 and SIRPαv2.

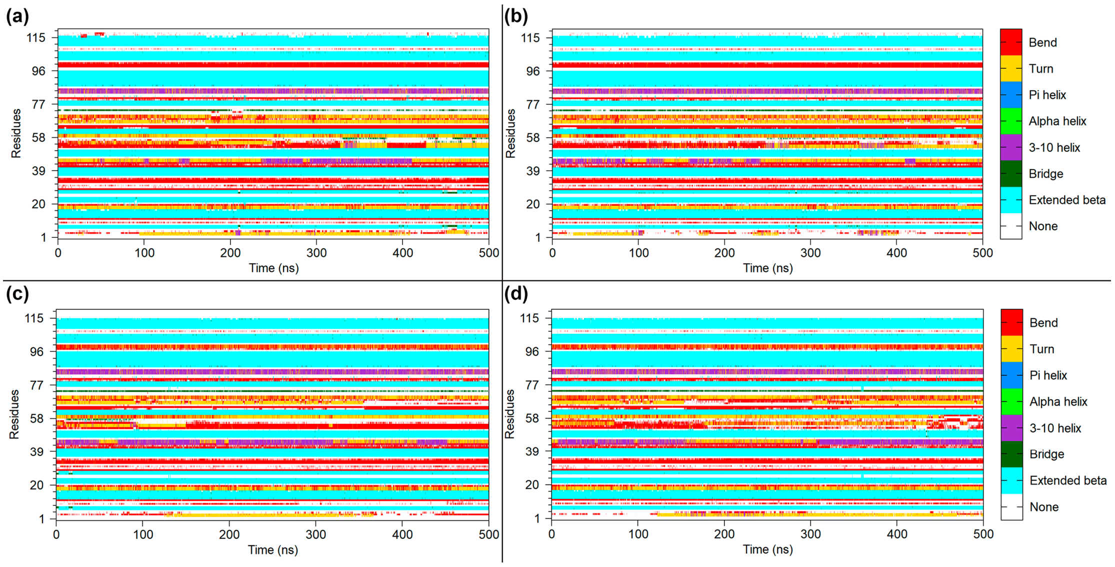

Figure S2. The DSSP analysis of SIRPα variants. (**a**) SIRPαv1 in simulation2, (**b**) SIRPαv1 in simulation3, (**c**) SIRPαv2 in simulation2, (**d**) SIRPαv2 in simulation3. The DSSP maps exhibit the secondary structure changes of SIRPαv1 and SIRPαv2 during simulations.

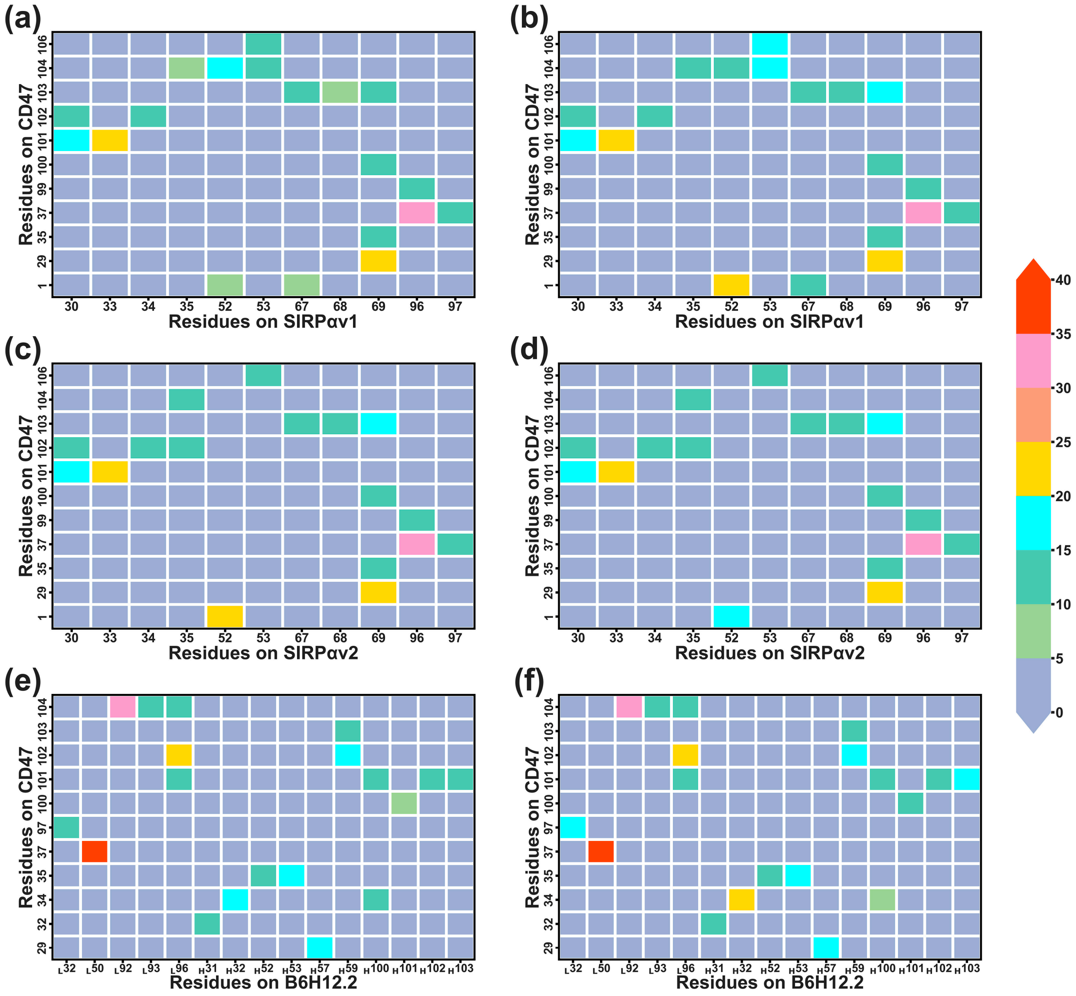

Figure S3. The residue-residue contact maps of three complexes. (**a**) CD47/SIRPαv1 in the second simulation, (**b**) CD47/SIRPαv1 in the third simulation, (**c**) CD47/SIRPαv2 in the second simulation, (**d**) CD47/SIRPαv2 in the third simulation, (**e**) CD47/B6H12.2 in the second simulation, and (**f**) CD47/B6H12.2 in the third simulation. The numerical values on the color bar represent fractions obtained by the native contact calculations. The fractions indicate the tightness of contacts between residues and the higher fractions represent the tighter contacts. The subscript "L" and "H" represent the light and the heavy chain, respectively.

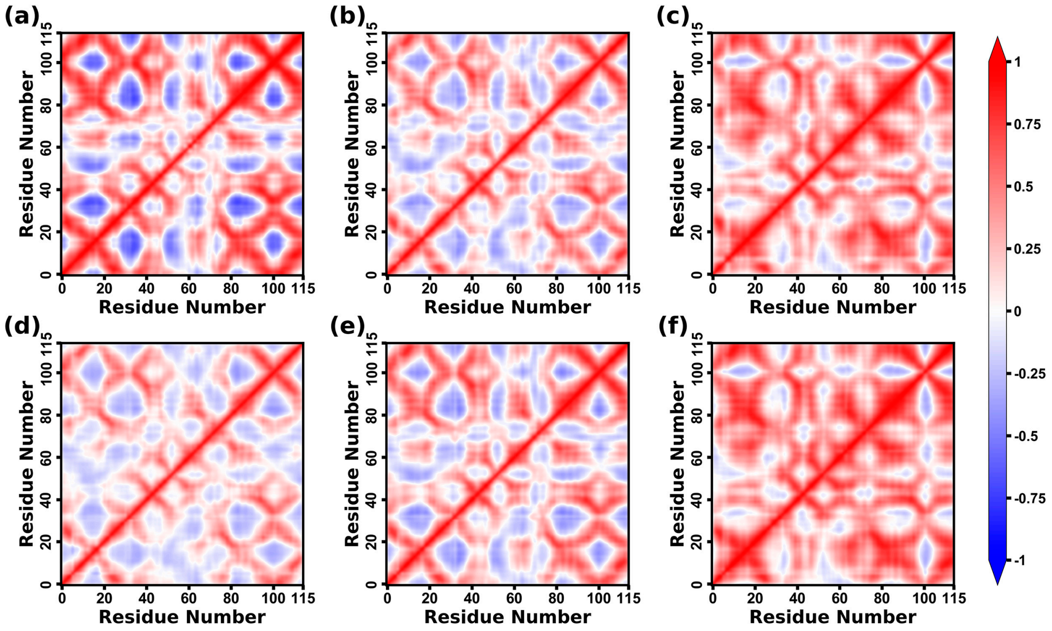

Figure S4. The Dynamical Cross Correlation Matrix (DCCM) maps for the CD47 proteins. (**a**) CD47/SIRPαv1 in the second simulation, (**b**) CD47/SIRPαv2 in the second simulation, (**c**) CD47/B6H12.2 in the second simulation, (**d**) CD47/SIRPαv1 in the third simulation, (**e**) CD47/SIRPαv2 in the third simulation, and (**f**) CD47/B6H12.2 in the third simulation. The motion correlations between Cα atoms were calculated by DCCM and different Cα atoms are used to represent the corresponding residues. The motion correlations are represented by values between -1 and +1, and the higher values indicate the stronger correlations. The maps on both sides of the DCCM diagonals are completely symmetrical.

Table S1. The Binding Free Energies for CD47 to the Binding Partners Calculated by MM-GBSA [a].

Contribution	CD47-SIRPαv1	CD47-SIRPαv2	CD47-B6H12.2
**Simulation2**			
ΔE_{ele}	-531.32 ± 107.31	-525.10 ± 53.86	-541.19 ± 29.78
ΔE_{vdw}	-84.12 ± 10.08	-84.39 ± 7.75	-98.18 ± 5.98
ΔE_{gas} [b]	-615.44 ± 114.34	-609.49 ± 55.75	-639.37 ± 29.64
$\Delta G_{sol\text{-}polar}$	551.85 ± 99.84	539.13 ± 50.65	557.91 ± 25.35
$\Delta G_{sol\text{-}np}$	-13.45 ± 1.89	-14.29 ± 0.92	-14.04 ± 0.61
ΔG_{sol} [c]	538.40 ± 98.14	524.84 ± 50.09	543.87 ± 25.14
ΔH_{tot} [d]	-77.05 ± 18.11	-84.65 ± 9.27	-95.50 ± 9.20
$T\Delta S$ [e]	-52.84 ± 9.54	-52.91 ± 6.57	-55.29 ± 7.32
ΔG_{bind} [f]	-24.21	-31.74	-40.21
**Simulation3**			
ΔE_{ele}	-614.16 ± 55.04	-498.05 ± 90.03	-542.20 ± 28.51
ΔE_{vdw}	-88.13 ± 6.93	-82.05 ± 8.83	-98.05 ± 6.05
ΔE_{gas}	-702.29 ± 55.88	-580.10 ± 93.72	-640.25 ± 28.68
$\Delta G_{sol\text{-}polar}$	626.16 ± 49.93	517.68 ± 83.19	559.28 ± 25.01
$\Delta G_{sol\text{-}np}$	-14.99 ± 0.74	-13.24 ± 1.42	-14.07 ± 0.56
ΔG_{sol}	611.17 ± 49.50	504.44 ± 82.08	545.21 ± 24.81
ΔH_{tot}	-91.13 ± 9.56	-75.66 ± 13.93	-95.04 ± 7.90
$T\Delta S$	-53.93 ± 5.77	-53.80 ± 6.49	-54.76 ± 6.34
ΔG_{bind}	-37.20	-21.86	-40.28

[a] The values after the "±" signs indicate the standard deviations. This table show the results of the second and the third simulations. The unit of Binding Free Energies is "*kcal/mol*".

[b] $\Delta E_{gas} = E_{internal} + E_{ele} + E_{vdw}$.

[c] $\Delta G_{sol} = E_{sol\text{-}np} + E_{sol\text{-}polar}$.

[d] $\Delta H_{tot} = \Delta E_{gas} + \Delta G_{sol}$, the enthalpy change.

[e] $T\Delta S$, entropy change.

[f] $\Delta G_{bind} = \Delta H_{tot} - T\Delta S$, the Gibbs free energy.

Table S2. The hydrogen bonds at the protein binding interfaces of CD47/SIRPαv1, CD47/SIRPαv2, and CD47/B6H12.2 [a].

Acceptor residues	Acceptor atoms	Donor residues	Donor atoms	Fraction1 [b]	Fraction2	Fraction3
**CD47-SIRPαv1:**						
SIRPα_Thr67	O	CD47_Arg103	NH1	88.39%	84.93%	91.16%
CD47_Leu101	O	SIRPα_Gly34	N	79.75%	73.11%	80.38%
CD47_Glu35	OE2	SIRPα_Arg69	NH2	49.75%	49.16%	52.81%
CD47_Glu35	OE1	SIRPα_Arg69	NH2	49.22%	50.90%	48.36%
CD47_Glu35	OE1	SIRPα_Arg69	NH1	34.85%	45.86%	43.36%
CD47_Glu35	OE2	SIRPα_Arg69	NH1	27.67%	41.40%	47.19%
CD47_Glu100	OE1	SIRPα_Arg69	NH1	31.02%	44.86%	37.69%
SIRPα_Gln52	O	CD47_Gln1	NE2	36.95%	7.24%	43.49%
CD47_Asp51	OD2	SIRPα_Arg95	NH1	27.76%	34.26%	32.49%
CD47_Glu100	OE2	SIRPα_Arg69	NH1	21.77%	24.67%	48.63%
CD47_Asp46	OD1	SIRPα_Ser98	OG	27.96%	27.41%	32.26%
CD47_Asp51	OD1	SIRPα_Arg95	NH1	28.08%	19.55%	31.52%
CD47_Asp46	OD2	SIRPα_Ser98	OG	20.27%	26.92%	33.74%
CD47_Asp51	OD2	SIRPα_Arg95	NH2	25.25%	27.88%	29.60%
CD47_Asp51	OD1	SIRPα_Arg95	NH2	25.86%	21.06%	28.09%
SIRPα_Gln52	OE1	CD47_Glu104	N	23.28%	21.12%	3.55%
**CD47-SIRPαv2:**						
SIRPα_Thr67	O	CD47_Arg103	NH1	86.75%	90.19%	85.80%
CD47_Leu101	O	SIRPα_Gly34	N	85.18%	83.24%	83.27%
CD47_Glu35	OE1	SIRPα_Arg69	NH2	50.61%	48.62%	52.99%
CD47_Glu35	OE2	SIRPα_Arg69	NH2	51.46%	52.00%	47.72%
CD47_Glu35	OE2	SIRPα_Arg69	NH1	51.63%	46.76%	49.83%
CD47_Glu100	OE1	SIRPα_Arg69	NH1	49.31%	45.71%	41.79%
CD47_Glu100	OE2	SIRPα_Arg69	NH1	37.97%	41.74%	45.84%
CD47_Glu35	OE1	SIRPα_Arg69	NH1	39.36%	44.43%	41.80%
CD47_Asp51	OD2	SIRPα_Arg95	NH1	28.26%	36.04%	24.54%
CD47_Asp51	OD1	SIRPα_Arg95	NH1	31.35%	30.61%	29.60%
CD47_Asp51	OD2	SIRPα_Arg95	NH2	28.00%	28.26%	23.61%
CD47_Asp51	OD1	SIRPα_Arg95	NH2	24.99%	29.48%	24.56%
**CD47-B6H12.2:**						
CD47_Thr99	OG1	LC_Hie92	NE2	77.36%	71.15%	67.44%
CD47_Glu100	O	HC_Gly102	N	76.87%	80.10%	80.90%
HC_Gly31	O	CD47_Thr34	OG1	70.27%	63.52%	68.58%
CD47_Asp51	OD2	LC_Gln53	NE2	62.09%	53.64%	36.49%
CD47_Glu104	OE1	LC_Arg96	NH1	55.32%	53.87%	41.68%
CD47_Leu101	O	LC_Arg96	NE	54.75%	55.88%	49.23%
CD47_Glu35	OE1	HC_Ser53	OG	52.38%	53.57%	34.55%
CD47_Glu35	OE2	HC_Ser53	OG	46.38%	45.29%	63.99%

CD47_Glu97	OE2	LC_Tyr32	OH	50.82%	41.96%	48.33%
CD47_Glu97	OE1	LC_Tyr32	OH	42.93%	50.97%	46.96%
CD47_Glu104	OE2	LC_Arg96	NH1	41.36%	44.13%	55.27%
CD47_Glu35	OE1	HC_Ser53	N	32.78%	35.26%	39.16%
CD47_Glu35	OE2	HC_Ser53	N	34.34%	39.36%	31.55%
CD47_Glu104	OE1	LC_Arg96	NH2	33.59%	33.64%	34.34%
CD47_Glu104	OE2	LC_Arg96	NH2	26.33%	30.08%	33.04%
CD47_Glu104	OE2	LC_Gly93	N	34.45%	28.24%	28.53%
CD47_Asp51 [c]	OD2	LC_Lys49	NZ	27.08%	25.98%	30.97%
CD47_Asp51 [d]	OD2	LC_Lys49	NZ	26.89%	26.95%	26.17%
CD47_Asp51 [e]	OD2	LC_Lys49	NZ	27.01%	22.54%	23.51%
CD47_Glu29	OE2	HC_Thr56	OG1	27.35%	23.70%	24.02%
CD47_Glu104	OE1	LC_Gly93	N	22.48%	28.45%	26.45%
CD47_Glu29	OE1	HC_Thr56	OG1	23.19%	18.54%	23.42%
HC_Ser53	OG	CD47_Asn32	ND2	21.81%	16.71%	20.89%

[a] The hydrogen bond interactions in three complexes. The hydrogen bond displayed in each entry appears in all of the triplicate and its fractions are greater than 20% in at least two replicates. LC: light chain, HC: heavy chain.

[b] The "Fraction" means the fraction data in the n-th simulation. The fraction in each entry is a percentage representing the time occupancy of corresponding hydrogen bond over the entire simulation.

[c] The donor hydrogen atom is HZ1 of Lys49 in B6H12.2.

[d] The donor hydrogen atom is HZ2 of Lys49 in B6H12.2.

[e] The donor hydrogen atom is HZ3 of Lys49 in B6H12.2.

Table S3. The residue contacts between CD47 and the binding partners of SIRPαv1, SIRPαv2, and B6H12.2 [a].

Partner Residues	CD47 Residues	Fraction1 (contact number) [b]	Fraction2 (contact number)	Fraction3 (contact number)
**CD47-SIRPαv1:**				
**SIRPαv1**	**CD47**			
Lys96	Tyr37	33.0091 (72)	33.4749 (70)	34.2263 (68)
Arg69	Glu29	22.3867 (75)	21.4958 (74)	20.2813 (73)
Val33	Leu101	20.8016 (44)	20.3648 (45)	20.6121 (45)
Arg69	Arg103	19.6945 (49)	13.7356 (53)	19.0957 (46)
Lys53	Glu106	18.0323 (61)	10.0193 (60)	15.3376 (64)
Gln52	Glu104	17.9137 (77)	16.0877 (75)	14.1651 (76)
Leu30	Leu101	16.8555 (51)	16.4331 (52)	17.7050 (49)
Gln52	Gln1	16.7226 (69)	8.3985 (64)	22.9481 (66)
Arg69	Glu100	16.2456 (44)	11.4590 (41)	13.6959 (40)
Lys53	Glu104	15.9955 (53)	14.6090 (72)	17.4360 (61)
Pro35	Glu104	13.1420 (33)	9.8040 (36)	13.6731 (35)
Thr67	Arg103	12.7309 (30)	14.9226 (54)	12.2632 (24)
Thr67	Gln1	12.5711 (56)	8.0386 (59)	12.5687 (56)
Leu30	Thr102	12.4768 (38)	11.1327 (37)	14.3165 (38)
Gly34	Thr102	12.4592 (28)	13.3457 (28)	13.1057 (26)
Gly97	Tyr37	12.3276 (26)	11.2895 (25)	12.3270 (26)
Arg69	Glu35	12.1039 (22)	12.8249 (19)	12.7934 (19)
Lys68	Arg103	11.1682 (30)	8.1097 (37)	12.6176 (30)
Lys96	Thr99	10.8717 (21)	10.3285 (22)	10.4585 (22)
**CD47-SIRPαv2:**				
**SIRPαv2**	**CD47**			
Lys96	Tyr37	32.6041 (70)	33.8034 (70)	32.9416 (69)
Val33	Leu101	20.5854 (43)	20.6529 (44)	20.6855 (43)
Arg69	Glu29	20.3434 (75)	21.6205 (75)	21.9778 (75)
Leu30	Leu101	17.3682 (50)	17.7394 (47)	17.4653 (47)
Arg69	Arg103	16.7920 (47)	17.4220 (44)	16.4169 (46)
Lys53	Glu106	15.2269 (69)	14.4445 (66)	10.3368 (67)
Gln52	Gln1	14.4663 (69)	22.5799 (70)	15.5595 (72)
Leu30	Thr102	13.8465 (36)	14.5438 (37)	13.7198 (36)
Arg69	Glu100	13.5221 (42)	13.9531 (40)	13.8270 (42)
Arg69	Glu35	13.0848 (20)	13.0813 (19)	13.2008 (20)
Gly34	Thr102	13.0199 (26)	13.3300 (27)	13.3222 (28)
Lys68	Arg103	12.8664 (32)	13.0592 (29)	13.3757 (32)
Pro35	Glu104	12.8312 (34)	13.6209 (35)	11.7165 (36)
Gly97	Tyr37	12.6846 (25)	12.3784 (24)	12.4533 (27)
Thr67	Arg103	12.1111 (38)	11.9762 (36)	11.5455 (34)
Pro35	Thr102	10.6633 (35)	10.8817 (36)	10.8957 (36)

Lys96	Thr99	10.6250 (22)	10.4250 (22)	10.2172 (23)
**CD47-B6H12.2:**				
**B6H12.2**	**CD47**			
LC_Phe50	Tyr37	36.1970 (60)	37.3950 (57)	37.6847 (58)
LC_Hie92	Glu104	31.4366 (59)	31.2090 (59)	31.3444 (58)
LC_Arg96	Thr102	21.2909 (48)	22.0511 (48)	21.4896 (48)
HC_Tyr32	Thr34	20.2062 (44)	19.8400 (45)	20.0178 (47)
HC_Tyr59	Thr102	19.0504 (61)	19.2135 (62)	18.6948 (63)
HC_Ser53	Glu35	16.2749 (27)	16.1168 (24)	16.1139 (22)
LC_Tyr32	Glu97	14.9205 (30)	14.9640 (30)	15.2287 (31)
HC_Asn103	Leu101	13.5196 (47)	13.9190 (47)	15.5076 (47)
HC_Tyr57	Glu29	13.4527 (82)	16.8604 (83)	16.1434 (87)
HC_Gly31	Asn32	13.3291 (25)	12.7699 (22)	12.8637 (23)
LC_Arg96	Glu104	11.9179 (22)	12.1761 (23)	12.0892 (23)
HC_Leu100	Leu101	11.3388 (29)	10.8494 (26)	12.0246 (26)
HC_Leu100	Thr34	10.9815 (39)	10.8422 (38)	9.8038 (36)
HC_Thr52	Glu35	10.7359 (25)	11.1753 (26)	11.3602 (25)
HC_Gly102	Leu101	10.7352 (22)	10.9733 (23)	11.0078 (23)
HC_Ala101	Glu100	10.5964 (19)	9.5177 (18)	10.3248 (19)
HC_Tyr59	Arg103	10.5198 (47)	10.8664 (41)	11.5413 (50)
LC_Arg96	Leu101	10.3549 (17)	10.6934 (17)	10.3312 (18)
LC_Gly93	Glu104	10.0974 (16)	10.1768 (17)	10.0075 (18)

[a] The residue pair displayed in each entry meets the criterions that the same residue pair formed contacts (distance within 5 $\mathring{A}$ in at least one frame) in all of the triplicate and its final fractions are greater than 10 in at least two replicates. LC: light chain, HC: heavy chain.

[b] The "Fraction" means the fraction data in the n-th simulation. The contact number in each bracket means total number of the atom pairs forming contacts within the corresponding residue pair.

Table S4. Comparisons of the Energy Decomposition with the mutation experimental data [a].

Residue	Mutation effect [b]	Decomposition value of the first simulation	Decomposition value of the second simulation	Decomposition value of the third simulation
**CD47** [c]				
E11K	low	0.005 +/- 0.013	0.015 +/- 0.011	0.017 +/- 0.011
C15G	low	-0.000 +/- 0.001	-0.000 +/- 0.001	-0.000 +/- 0.001
V20D	low	0.004 +/- 0.001	0.004 +/- 0.001	0.004 +/- 0.001
Y37D	high	-3.607 +/- 0.629	-3.646 +/- 0.600	-3.535 +/- 0.640
D46K	high	0.092 +/- 0.733	0.068 +/- 0.732	0.091 +/- 0.685
A53K	low	-0.281 +/- 0.194	-0.292 +/- 0.187	-0.302 +/- 0.178
K67E	low	0.071 +/- 0.022	0.063 +/- 0.021	0.061 +/- 0.024
Q72K	low	0.016 +/- 0.008	0.017 +/- 0.008	0.017 +/- 0.008
E97K	high	-1.102 +/- 1.576	-3.038 +/- 1.729	-0.866 +/- 1.768
E100K	high	-2.151 +/- 0.917	-2.148 +/- 0.893	-2.007 +/- 0.894
E106K	middle	-0.785 +/- 1.276	-0.577 +/- 1.792	-0.507 +/- 1.424
**SIRPαv1** [d]				
E2D	low	0.218 +/- 0.044	0.292 +/- 0.119	0.317 +/- 0.121
D10E	low	0.137 +/- 0.011	0.155 +/- 0.011	0.142 +/- 0.010
T26A	low	0.022 +/- 0.012	0.017 +/- 0.012	0.017 +/- 0.012
A27M	high	0.012 +/- 0.023	0.012 +/- 0.016	-0.003 +/- 0.021
Q37M	high	0.048 +/- 0.383	0.022 +/- 0.383	-0.106 +/- 0.760
P44A	low	0.003 +/- 0.005	0.002 +/- 0.005	0.003 +/- 0.005
M72L	medium	-0.054 +/- 0.038	-0.041 +/- 0.035	-0.061 +/- 0.043
**SIRPαv2** [e]				
D10K	low	0.142 +/- 0.011	0.143 +/- 0.011	0.148 +/- 0.012
V27M	high	-0.123 +/- 0.066	-0.105 +/- 0.047	-0.099 +/- 0.056
I31K	high	-0.958 +/- 0.293	-1.171 +/- 0.326	-1.161 +/- 0.366
V33E	high	-6.032 +/- 0.547	-5.948 +/- 0.526	-5.934 +/- 0.536
Q37E	medium	0.002 +/- 0.658	-0.584 +/- 1.514	-0.535 +/- 1.515
P44R	low	0.003 +/- 0.003	0.004 +/- 0.004	0.004 +/- 0.004
E54K	low	-0.164 +/- 0.711	1.483 +/- 0.741	0.256 +/- 0.975
H56D	medium	-0.609 +/- 1.117	0.082 +/- 0.138	-0.084 +/- 0.700
S66D	high	-0.329 +/- 1.125	-0.978 +/- 1.370	-0.568 +/- 1.202
R69E	high	-13.463 +/- 1.681	-13.754 +/- 1.824	-13.849 +/- 1.859
E70K	low	0.889 +/- 0.461	0.849 +/- 0.459	0.880 +/- 0.505
M72R	low	-0.043 +/- 0.037	-0.043 +/- 0.032	-0.039 +/- 0.029
V92E	medium	0.027 +/- 0.016	0.031 +/- 0.015	0.029 +/- 0.016
F94R	high	-0.029 +/- 0.060	-0.006 +/- 0.056	-0.016 +/- 0.057
K96E	high	-8.090 +/- 2.913	-9.454 +/- 3.051	-6.700 +/- 3.391
S98R	medium	-1.499 +/- 1.213	-1.850 +/- 1.277	-1.548 +/- 1.252
D100K	high	1.421 +/- 1.274	0.707 +/- 2.332	1.116 +/- 1.765
T101K	low	0.083 +/- 0.051	0.087 +/- 0.052	0.085 +/- 0.051

F103D	medium	0.010 +/- 0.019	-0.004 +/- 0.022	0.002 +/- 0.024
R110K	low	0.115 +/- 0.008	0.117 +/- 0.008	0.119 +/- 0.009

[a] The unit of Energy decomposition is "*kcal/mol*"

[b] The mutation experiments [1,2] indicated that affinities of residues changed to less than 35% of natural states after mutation had the high mutation effects, residues changed to 35% and 65% had the middle mutation effects, and residues changed to more than 65% had the low mutation effects.

[c] The mutation experimental data for CD47 in CD47/SIRPαv2 is cited from Hatherley et al. [1].

[d] The mutation experimental data for SIRPαv1 is cited from Liu et al. [2].

[e] The residue mutation experimental data for SIRPαv2 is cited from Hatherley et al. [1] and Liu et al. [2].

References

1. Hatherley, D.; Harlos, K.; Dunlop, D. C.; Stuart, D. I.; Barclay, A. N., The structure of the macrophage signal regulatory protein alpha (SIRPalpha) inhibitory receptor reveals a binding face reminiscent of that used by T cell receptors. *J Biol Chem* **2007,** 282, (19), 14567-75.
2. Liu, Y.; Tong, Q.; Zhou, Y.; Lee, H.-W.; Yang, J. J.; Bühring, H.-J.; Chen, Y.-T.; Ha, B.; Chen, C. X. J.; Yang, Y.; Zen, K., Functional Elements on SIRPα IgV Domain Mediate Cell Surface Binding to CD47. *Journal of Molecular Biology* **2007,** 365, (3), 680-693.
